# Effectiveness of technology-enhanced teaching methods of undergraduate dental skills for local anaesthesia administration during COVID-19 era: students’ perception

**DOI:** 10.1186/s12903-022-02077-6

**Published:** 2022-02-13

**Authors:** Rasa Mladenovic, Sakher AlQahtani, Kristina Mladenovic, Zoran Bukumiric, Sobia Zafar

**Affiliations:** 1 Department of Dentistry, Faculty of Medical Sciences, University of Kragujevac, Kragujevac, Serbia; 2 College of Dentistry, King Saud University, Riyadh, Saudi Arabia; 3 Center for Rehabilitation Medicine, University Clinical Center Kragujevac, Faculty of Medical Sciences, University of Kragujevac, Kragujevac, Serbia; 4 Institute of Medical Statistics and Informatics, School of Medicine, University of Belgrade, Belgrade, Serbia; 5 School of Dentistry, The University of Queensland, Brisbane, Australia

**Keywords:** Augmented reality, e-Learning, Serious games, Simulation, 3D, Local anaesthesia, COVID-19

## Abstract

**Background:**

Traditional face-to-face clinical learning became problematic for final year dental students during the COVID-19 pandemic. Distance-learning may help mitigate the immediate impact of dental school closures. Integrating e-learning technologies into the learning process helps bridge the gap between pre-clinical and clinical training. Simulation allows students to repeat procedures until they demonstrate acceptable levels of skill. This study aimed to determine the effectiveness of a serious game as an additional teaching tool during the COVID-19 era to improve dental students’ local anaesthesia administration technique and confidence.

**Methods:**

This study applied a simulation-based serious game as an additional learning tool for training and educating dental students in local anaesthesia. Students used a mobile simulator in Serbian for 10 days from their homes. To evaluate the learning process, the students completed a post-training questionnaire.

**Results:**

All respondents felt comfortable using the simulator. Over 90% of respondents believed that the application facilitated the learning process and had advantages in terms of accessibility and ease of use. Also, students found augmented reality (AR) technology particularly interesting to use. The use of a mobile simulator designed as a 3D and AR environment allows for simpler localisation and identification of anatomical structures and reference points, which is a good base for clinical practice.

**Conclusion:**

Serious games of local anaesthesia procedures as an additional e-learning tool during the COVID-19 era could improve students’ knowledge and skills.

**Supplementary Information:**

The online version contains supplementary material available at 10.1186/s12903-022-02077-6.

## Background

Simulation is defined as a situation in which a particular set of conditions is created artificially to study or experience something that could exist in reality [1]. Today, simulation is used for various purposes, including entertainment, training, and education. It is commonly used in areas where people manipulate or control complex systems or procedures. Some simulators are designed to be as realistic as possible and provide an experience almost identical to a real system, while others reproduce only the key features in a simulated environment. Simulators are used when the use of the actual system is not practical due to life-threatening effects, financial constraints, or ethical considerations [2]. Obtaining local anaesthesia (LA) is a critically important skill in operative dentistry, therefore competence in this field is crucial for dental students.

After the COVID-19 pandemic, traditional teaching and learning methods became challenging globally for dental students. Although restrictive measures were relaxed during 2021, the number of patients who are an integral part of clinical training remained limited to prevent transmission by asymptomatic carriers, either students or patients. To bridge the gap between pre-clinical and clinical training, it is necessary to integrate e-learning technologies into the learning process [3, 4]. Over the past decade, serious games have become highly relevant in the fields of medical education and health promotion [5]. Serious games are those whose primary purpose is not entertainment, enjoyment, or fun; rather learning or practising a skill. They are defined as an interactive computer or mobile application, with or without a significant hardware component, that has a challenging goal, is fun to play, includes some scoring mechanism, and provides the user with improved skills, knowledge, or attitudes helpful in reality [6–9]. Three-dimensional (3D) environments, augmented reality (AR), virtual reality (VR) and building information modelling are the trending technologies used by researchers and developers in the field [10]. 3D technology can be used as a basis for simulations, to deliver authentic learning experiences that are difficult, impractical, or impossible to achieve in the real world [11]. Augmented Reality is one of the sophisticated learning methods, and it does not create a new virtual environment but complements the real world by adding virtual elements [4]. The study aimed to determine the effectiveness of the serious game as an adjunct teaching tool during the COVID-19 era in improving the dental student’s LA administration technique, experience, and confidence.

## Methods

### Study participants

**Fig. 1:**
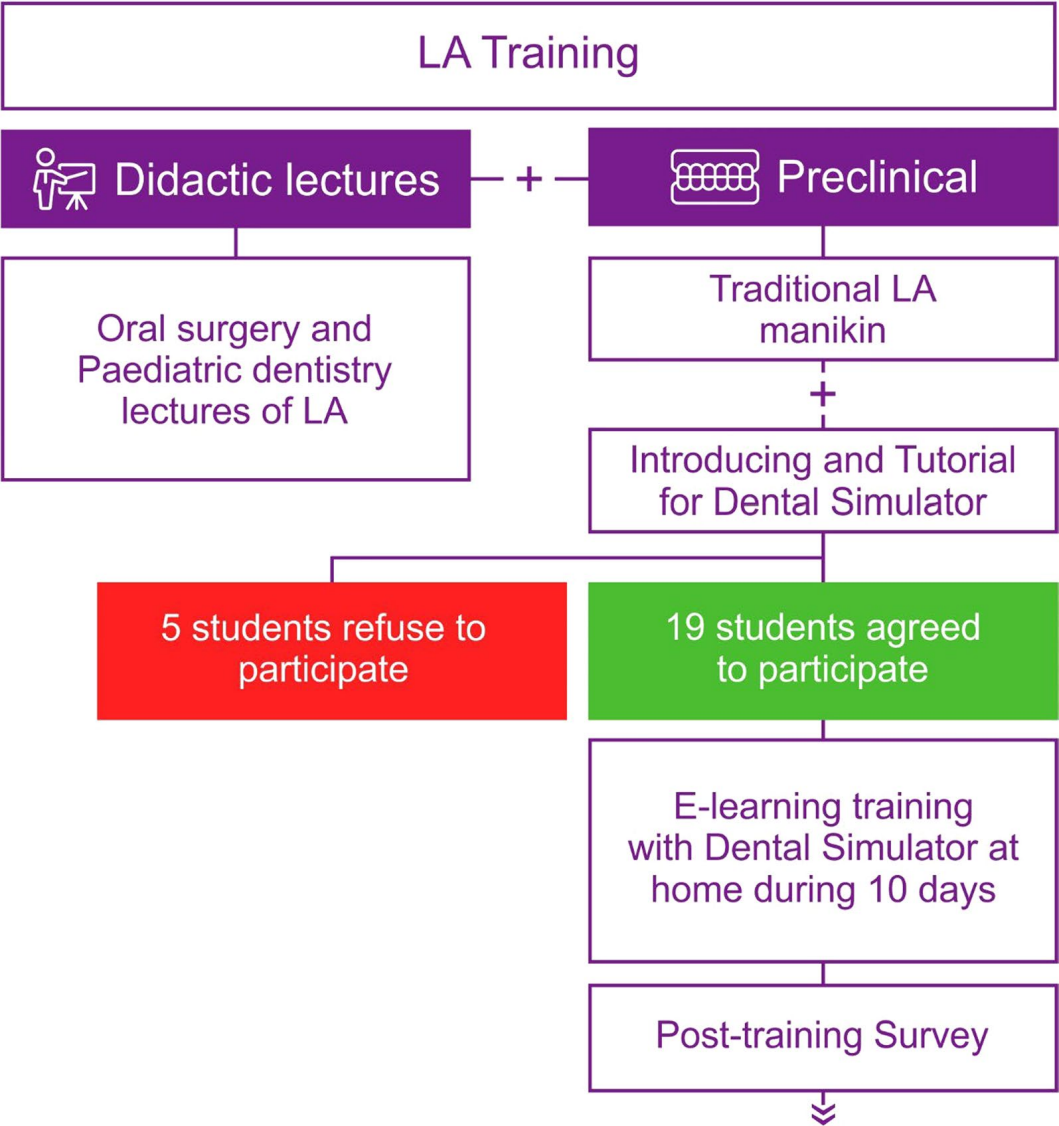
The flow diagram showing the study protocol

This study applied a simulation-based serious game as an adjunct learning tool for LA training to provide the best possible education to dental students during the COVID-19 era. After completing the conventional teaching for LA, including theoretical learning and hands-on experience on traditional LA manikin heads, all fourth-year students were invited to participate in this study using serious game LA simulation training. The LA learning curriculum and study design are given in the flow diagram (Fig. 1).

### Ethics approval

It was obtained from the Institutional Review Commission of the Faculty of Medicine, the University of Pristina in Serbia (Ethics Approval No. 09-1771-1).

### E-Learning platform

The study utilised a mobile simulator application (Dental Simulator v1.13 for iOS and Android). The participants used the application in Serbian, however, it is also available in English, Brazilian, and Spanish. The participants who agreed to participate were enrolled via the University mode of the application. The *‘University mode’* allowed to record the participants’ attempts and enable educators to visualise the recorded attempts of the participants at any time. All participants were provided with detailed operating instructions regarding application and were allowed to use it for ten days from their home. The Mobile simulator has three modes: Study mode, Simulation mode and Augmented Reality mode.

#### Study mode

**Fig. 2:**
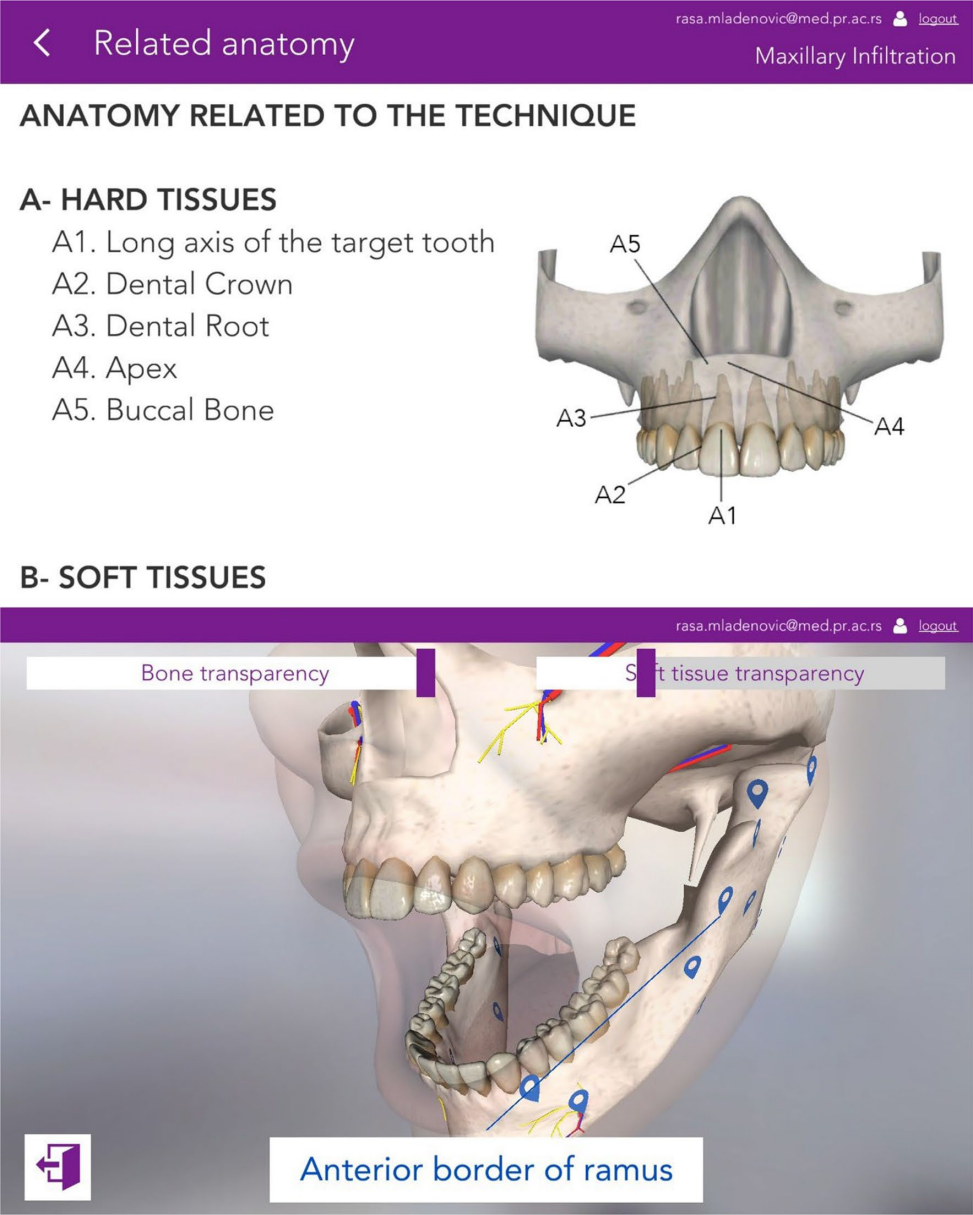
The study mode of the simulator demonstrating the anatomical features offered by the application

In this mode, users can read technical descriptions and watch clinical and simulation videos to acquaint themselves with LA’s technique better. In addition, this mode allows the users to study anatomical structures through 3D atlas. Furthermore, the option of deleting anatomical sections allows for visualising each structure in detail (Fig. 2).

#### Simulation (3D) mode

**Fig. 3:**
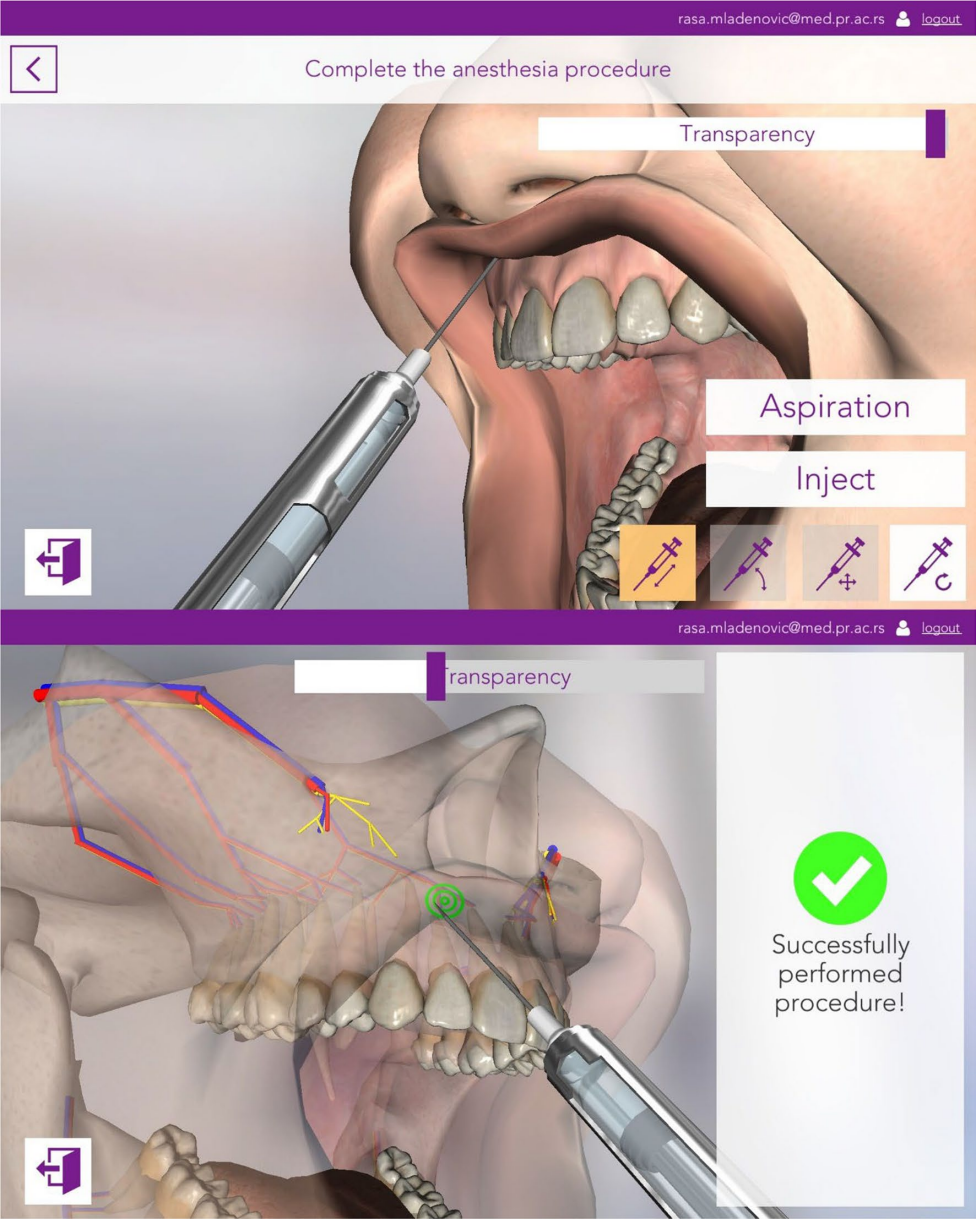
The figure demonstrating the syringe manipulation and successful performance of the operator for the maxillary infiltration simulation model

**Fig. 4:**
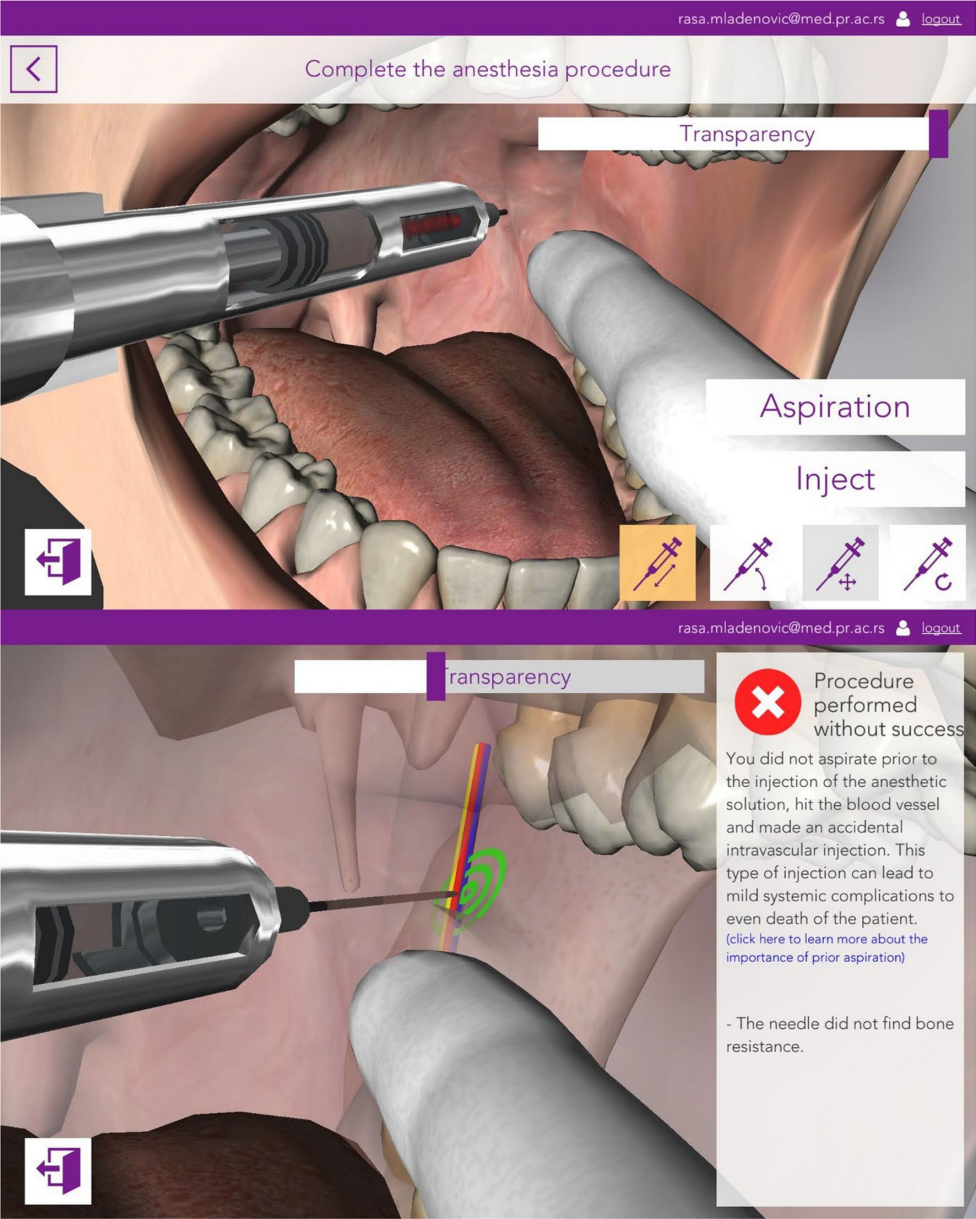
The figure demonstrating the blood aspiration and failed performance of inferior alveolar nerve block anaesthesia simulation model

This mode allows for simulation of LA in a 3D environment (Figs. 3 and 4). The users could delete the soft tissues of the oral cavity at any time, which allows them to better determine the exact location of the puncture for a particular LA technique. After completing each exercise, feedback is provided on whether it was a success or there were errors, so that users can reflect on their mistakes.

#### Augmented reality mode

**Fig. 5:**
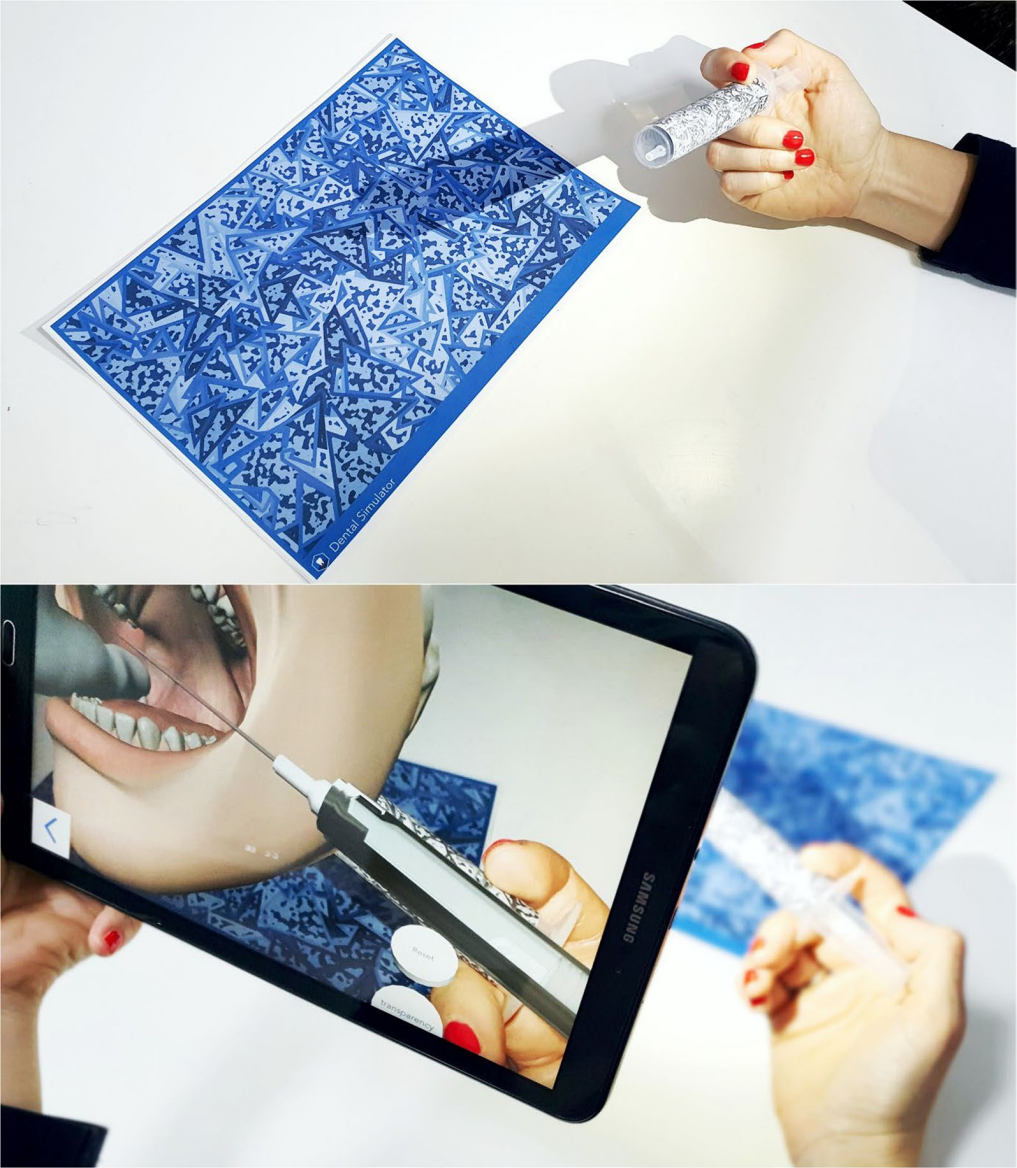
The figure demonstrating the use of AR simulator at home

Vuforia Engine-powered application (PTC, Parametric Technology Corporation, Boston, USA) brings immersive experience using the camera of a mobile device or Virtual Reality (VR) glasses. As image targets are the basis for image recognition technology, participants received printing instructions on how to print a virtual patient AR tracker on A4 paper, and stick the AR tracker on a 20 ml syringe (Fig. 5).

### Evaluation of the learning process

The participants completed a validated post-training survey to evaluate the learning process [12, 13]. The questionnaire aimed to determine dental students’ perceptions of experience gained through digital LA simulation compared to the traditional LA training. The questionnaire begins with asking the age and gender of the participant, type of device usage in everyday learning and time spent with mobile simulators during the study (Additional file 1: Appendix 1). The satisfaction questions that evaluated their knowledge and skills about LA procedures used a series of statements with five-point Likert Scale responses. When responding to a Likert item, respondents rank their level of agreement with a statement on a scale from 1 to 5: strongly disagree = 1; partially disagree = 2; neutral = 3; partially agree = 4; strongly agree = 5. Each respondent’s answer has scored accordingly, and then the responses are summed to create an aggregate score expressing the respondent’s critical attitude towards the subject. The questionnaires were administered online using Google forms (Google.com, Mountain View, California, United States).

### Statistical analysis

The data were tabulated on Microsoft Excel (Version 16.30, Microsoft Corp., Redmond, Washington) spreadsheet and then processed in the R software environment (R Core Team, 2019) for descriptive analysis. The data were presented as number (n) and percentage (%) or mean ± standard deviation. The Wilcoxon one-sample test was used to test whether the sample’s median is equal to a known standard value (3-neutral). Linear regression was used to model the relationship between the dependent variable (total score for all questions) and potential predictors (gender, age, period of usage, experience with anaesthesia and device usage). Statistical hypotheses were tested at the level of statistical significance of 0.05.

## Results

All students enrolled in the fourth year of their dental degree (n = 24) were invited to participate in the study, however, 19 participants agreed to participate in the study. Thus the response rate of the survey was 79.1%. Of the total participants, 9 (47%) were female, and 10 (53%) were male. The mean age of the participants was 23 years. Almost all students used smartphones (90%) and laptops (95% of respondents) daily. More than half of the participants spent more than six hours practising through simulation (53%) during the ten-day**.** Of the maximum value of 55 for the total score, the mean value was 45 ± 5, the minimum value was 35, and the maximum was 53. Subjects with experience in administering anaesthesia to an actual patient had significantly higher total scores (b = 6.229; *p* = 0.040). Gender, age and device usage were not associated with a higher overall score. A longer usage period was associated with higher scores but did not reach statistical significance (b = 2.198; *p* = 0.081) (Table 1).

**Table 1:** Descriptive data showing the participants demographics and their time spend on the device usage and the linear regression analysis with total score as a dependent variable

Variables	n (%)	Linear regression	
		b	*p*
**Gender**		−0.589	0.803
Male	10 (53)	–	–
Female	9 (47)	–	–
**Age**		−0.091	0.954
**Experience with local anaesthesia administration**	3 (16)	6.229	0.040
**Device usage in everyday learning**			
Smartphone	17 (90)	−3.265	0.389
Laptop	18 (95)	−0.444	0.933
Tablet		−3.267	0.248
**Spent time with mobile simulator during study**	4 (21)	2.198	0.081
3 h and less	6 (32)	–	–
3–6 h	3 (16)	–	–
More than 6 h	10 (53)	-	–

All respondents (100%) felt comfortable using the simulator. Over 90% of them believed that the application facilitated the learning process and had an advantage in terms of accessibility and ease of use. Also, students found AR technology particularly interesting to use (89.5%). The median score for all statements was significantly higher than the neutral value (*p* < 0.05) (Table 2).

**Table 2:** The response of the participants regarding their satisfaction with the use of application

Statements	Five-point likert scale responses					
	Strongly disagree n (%)	Partially disagree n (%)	Neutral n (%)	Partially agree n (%)	Strongly agree n (%)	*p*-value
I felt comfortable using the LA dental simulator	0 (0)	0 (0)	2 (11)	10 (53)	7 (37)	< 0.001
Local anaesthesia dental simulator is user friendly	0 (0)	0 (0)	3 (15.8)	8 (42.1)	8 (42.1)	< 0.001
3-D images of the anatomy in the LA dental simulator looked realistic	0 (0)	0 (0)	3 (15.8)	8 (42.1)	8 (42.1)	< 0.001
Using LA dental simulator assisted my learning	0 (0)	0 (0)	2 (10.5)	9 (47.4)	8 (42.1)	< 0.001
I felt more confident about my LA administration skills after using LA dental simulator	1 (5.3)	1 (5.3)	5 (26.3)	8 (42.1)	4 (21.1)	0.021
3-D anatomical structures on LA dental simulator improved my understanding of anatomical landmarks	0 (0)	0 (0)	3 (15.8)	8 (42.1)	8 (42.1)	< 0.001
The use of LA dental simulator added value in my training compared to relying solely on traditional methods of training	1 (5.3)	1 (5.3)	3 (15.8)	11 (57.9)	3 (15.8)	0.010
The use of LA dental simulator improved my skills of LA administration	0 (0)	4 (21.1)	3 (15.8)	8 (42.1)	4 (21.1)	0.022
When using LA dental simulator, I felt I was engaged in a learning activity	0 (0)	0 (0)	2 (10.5)	10 (52.6)	7 (36.8)	< 0.001
I found AR particularly interesting to use	0 (0)	0 (0)	2 (10.5)	8 (42.1)	9 (47.4)	< 0.001
I think the use of LA dental simulator would be helpful in teaching LA administration technique	0 (0)	1 (5.3)	1 (5.3)	7 (36.8)	10 (52.6)	< 0.001

## Discussion

The findings indicate a positive effect of simulation-based serious games used in this study on learning LA in the study population. The serious games for learning LA can provide interactive ways of presenting materials and therefore should be used as an adjunct learning tool, especially within e-learning environments. This additional learning tool could help students develop practice skills and capabilities, e.g., problem-solving, critical thinking, clinical skills, and apply them in authentic professional contexts (simulation, role play, virtual). The challenges in providing face-face teaching in this rapidly changing Covid-19 era, the simulator-based serious games provide an alternative, economic and flexible learning environment. The administration of profound LA is a key skill for dentists and a prerequisite for most dental treatments. Thus, the use of simulation-based LA skills could allow the student to continue developing their professional identity, decision-making and judgement related to LA administration. Moreover, the students continue to achieve the subject learning outcome and consequently can replicate authenticity in the workplace environment.

The significance of this technology is that it allows for the 3D planning of a procedure. In recently published studies, Mladenovic et al. examined the effect of using the same mobile simulator in clinical practice [14–16]. The results showed that students who used AR simulation mode were better acquainted with anatomical details and reference points for inferior alveolar nerve block anaesthesia. In addition, this group reported better syringe manipulation and greater anaesthetic success compared to students who received conventional training [14–16]. As a mobile device is the only pre-requisite for successful training, the main advantage of this type of simulation (serious game) is the ease of access, so that students can study at their own pace without supervision [15, 17]. This simulation method can also allow students to practice in a safe space themselves and others, e.g., patients, students can make mistakes as part of the learning process, but without any harm to patients. Exploring these actions and their consequences provides rich learning experiences and is further enhanced by careful design of accompanying activities to introduce the simulation or unpack the learning afterwards e.g., with peers or the instructor.

The study showed that participants spend up to five hours per day on their mobile devices, whereas they allocate two hours daily on so-called unnecessary content, including social networks, games and such [14]. This data demonstrated the importance of investing effort and resources in developing high quality digital educational content using serious games. Although the study highlighted the importance of serious games designed to meet the needs of the learners, it could also help them in utilising their time for educational purposes rather than entertainment purposes. It is crucial to keep up with the current digital era and embed digital learning tools within traditional teaching, and to involve experienced lecturers and educators in this process. This would provide students with innovative educational tools. The part of time they spend on the entertainment games could be replaced with serious games, which could enhance learning and productivity. As serious games could act as entertaining tools with a purpose of education, thus it has the potential to replace the time spent on the “entertainment games” with the’serious games’. This could act as a continuous educational tool improving the quality of learning. The language barrier is a common cause of problems with using educational applications as most of these cater to English, German and Spanish speakers. There has been a continuous effort to translate the educational applications in the native language of the users to enhance their utility. The current tool provided the user interface in participants’ native language, Serbian, thus enhancing the application usage without language difficulties.

One of the limitations of the study was the small sample size, however it was conducted as a pilot study and the authors plan to undertake large scale similar studies in the future. Furthermore, the main challenge of simulation in an AR environment is image tracker detection which requires well-lighted space and gentle manipulation of the device. The application detection of image tracker could be improved by upgrading the detection system and incorporating additional detection sensors. Also, enhanced utilisation of the haptic (that allows the device to vibrate when the needle comes in contact with the bone) could be employed in the future. Furthermore, more realistic anatomical details, involuntary movements of the patient, and the option of choosing the patient’s age could also be incorporated. This feature is critical in managing inferior alveolar nerve block in children, where the position of the mandibular foramen differs significantly from that of adults.

## Conclusion

The results of this pilot study demonstrated that simulation-based serious games for LA procedures could be used as an adjunct e-learning tool for the participants during the COVID19 era and beyond. Furthermore, it could enhance the knowledge and skills for the LA procedures of the participants, which are the pre-requisite of many dental procedures.

## Supplementary Information

**Additional file 1**. Appendix 1: Questionnaire

## Data Availability

The datasets generated and/or analysed during the current study are not publicly available as ethics approval was granted on the basis that only the researchers involved in the study could access the identified data but are available from the corresponding author on reasonable request.

## Abbreviations

LA: Local anesthesia
AR: Augmented Reality
VR: Virtual Reality
3D: Three-dimensional environment
