# Supplementary material for: Effectiveness of technology-enhanced teaching methods of undergraduate dental skills for local anaesthesia administration during COVID-19 era: students’ perception
Source: BMC Oral Health. 2022 Feb 13;22:40. doi: 10.1186/s12903-022-02077-6 (PMC8842892; doi:10.1186/s12903-022-02077-6)
Supplement: Supplementary file 1 — Additional file 1. Appendix 1: Questionnaire [file 12903_2022_2077_MOESM1_ESM.pdf]

Effectiveness of technology-enhanced teaching methods of undergraduate dental skills for local anaesthesia administration during COVID-19 era: Students' perception

Instructions: Please tick the appropriate box or write your answer on the dotted line:

SECTION A: DEMOGRAPHIC INFORMATION

1. What is your gender?

☐ Male
☐ Female

2. What is your age? ………………………………………………………………………………………… (Years)

3. What is your undergraduate Year level?

☐ Year 2
☐ Year 3
☐ Year 4
☐ Year 5

4. Do you use any of the following regularly?

☐ Smartphone
☐ Tablet/iPad
☐ Laptop

5. Spent time with mobile simulator during study

☐ 3 hours and less
☐ 3-6 hours
☐ more than 6 hours

6. Experience with local anaesthesia administration:

☐ Yes
☐ No

SECTION B- LOCAL ANAESTHESIA SIMULATOR

1. I felt comfortable using the LA dental simulator

Strongly Disagree	Disagree	Neutral	Agree	Strongly Agree
☐	☐	☐	☐	☐

2. Local anaesthesia dental simulator is user friendly

Strongly Disagree	Disagree	Neutral	Agree	Strongly Agree
☐	☐	☐	☐	☐

3. 3-D images of the anatomy in the LA dental simulator looked realistic

Strongly Disagree	Disagree	Neutral	Agree	Strongly Agree
☐	☐	☐	☐	☐

4. Using LA dental simulator assisted my learning

Strongly Disagree	Disagree	Neutral	Agree	Strongly Agree
☐	☐	☐	☐	☐

5. I felt more confident about my LA administration skills after using LA dental simulator

Strongly Disagree	Disagree	Neutral	Agree	Strongly Agree
☐	☐	☐	☐	☐

6. 3-D anatomical structures on LA dental simulator improved my understanding of anatomical landmarks

Strongly Disagree	Disagree	Neutral	Agree	Strongly Agree
☐	☐	☐	☐	☐

7. The use of LA dental simulator added value in my training compared to relying solely on traditional methods of training

Strongly Disagree	Disagree	Neutral	Agree	Strongly Agree
☐	☐	☐	☐	☐

8. The use of LA dental simulator improved my skills of LA administration

Strongly Disagree	Disagree	Neutral	Agree	Strongly Agree
☐	☐	☐	☐	☐

9. When using LA dental simulator, I felt I was engaged in a learning activity

Strongly Disagree	Disagree	Neutral	Agree	Strongly Agree
☐	☐	☐	☐	☐

10. I found AR particularly interesting to use

Strongly Disagree	Disagree	Neutral	Agree	Strongly Agree
☐	☐	☐	☐	☐

11. I think the use of LA dental simulator would be helpful in teaching LA administration technique

Strongly Disagree	Disagree	Neutral	Agree	Strongly Agree
☐	☐	☐	☐	☐

……….

Thank you for completing this survey.
